# Using IFN-gamma release assay to confirm tuberculin skin test improves the screening of latent tuberculosis infection in Italian healthcare workers

**DOI:** 10.1186/s12995-016-0117-6

**Published:** 2016-06-07

**Authors:** Raffaela Olivieri, Sara Scarnera, Annalisa Ciabattini, Giulia De Vuono, Pietro Manzi, Gianni Pozzi, Giuseppe Battista, Donata Medaglini

**Affiliations:** Laboratorio di Microbiologia Molecolare e Biotecnologia, Dipartimento di Biotecnologie Mediche, Università degli Studi di Siena, Siena, Italy; Medicina Preventiva e Sorveglianza Sanitaria, Dipartimento di Biotecnologie Mediche, Universita’ degli Studi di Siena, Siena, Italy; Direzione Medica di Presidio, Azienda Ospedaliera Universitaria Senese, Siena, Italy

**Keywords:** LTBI, Tuberculosis, Interferon-gamma release assays, Healthcare workers

## Abstract

**Background:**

Healthcare workers (HCWs) represent a tuberculosis (TB) risk group for a wide range of tasks in healthcare, even in countries with low TB incidence, like Italy. Latent Tuberculosis Infection (LTBI) screening programs are an important tool for TB prevention in these setting.

**Methods:**

A retrospective study under a LTBI screening program among HCWs at the Siena University Hospital (Italy), was conducted between September 2011 and July 2015. Tuberculin Skin Test (TST) was used as a first level examination; all TST-positive cases were tested with QuantiFERON-TB Gold In-Tube (QFT-GIT) test, together with a group of TST-negative subjects.

**Results:**

Among the 2136 HCWs screened, 144 (6.7 %) were TST-positive and therefore tested with QFT-GIT, confirming a positive result in 36 cases (25 %). Agreement between two tests was poor (*k* = 0.092; 95 %, Confidence Interval [CI]- 0.048–0.136, *p* = 0.002). Among TST-positive cases, discordant results occurred more frequently in BCG vaccinated than unvaccinated HCWs (86.3 %, *p* < 0.001). The probability of a QFT-GIT-positive result increased according to the TST diameter (*p* = 0.001). No putative risk factor was associated with LTBI occurrence.

**Conclusions:**

The use of QFT-GIT test as a second step in TST-positive cases offers an appropriate tool for LTBI detection, especially among BCG-vaccinated HCWs.

## Background

Healthcare workers (HCWs) are an important group at increased risk for exposure to various infectious agents including *Mycobacterium tuberculosis*. Probability of exposure to tuberculosis (TB) infection depends on the specific tasks and settings; for this reason HCWs are stratified in different risk levels and should be included in TB screening programs [1, 2].

Systematic testing for diagnosis of Latent Tuberculosis Infection (LTBI) is an important component of infection control strategies among HCWs [3]. LTBI is a state of immune response to stimulation by *M. tuberculosis* antigens without evidence of clinically manifested active TB [3]. The lifetime risk of TB reactivation in case of LTBI is estimated to be 5–10 %, decreasing over the years depending on several factors, the most important one being the immunological status of the host [3, 4]. In many high-income countries, periodic screening of HCWs and contacts of confirmed TB patients for LTBI is a routine component of TB control, including routinely repeated screening or tracing after accidental contact with infected patients or materials.

Until the introduction of Interferon Gamma Release Assays (IGRAs), the only method for LTBI detection was Tuberculin Skin Test (TST). TST is based on an intradermal injection of a purified protein derivative (PPD) from *M. bovis* to elicit T-cell mediated delayed-type hypersensitivity reaction if the person has been previously infected with *M. tuberculosis*. However, TST assay has several intrinsic limitations: i) low sensitivity; ii) subjective interpretation of results; iii) cross reaction with Bacillus Calmette-Guerin (BCG) vaccination and non-tuberculous mycobacteria (NTM) infections iv) booster phenomenon [5–8]. The interpretation of TST results in the serial testing of HCWs is a major issue: findings show TST reversions or unclear conversions resulting from either random variability (e.g., differences in administration, reading or biologic response), boosting effect or an actual new infection [9–12].

IGRAs overcome some of these shortcomings and have emerged as an alternative to TST. IGRAs are indeed ex-vivo blood-based tests, are not affected by BCG vaccination status and by most infections with environmental NTM, can be repeated any number of times without sensitization or boosting, require only one patient visit and the result is available within 24 h.

To date, there are two commercial IGRAs, the QuantiFERON®-TB Gold In-Tube (QFT-GIT, QIAGEN, Hilden, Germany) test that measures by ELISA assay the amount of IFN-γ released by circulating T cells and the T-SPOT®.*TB* assay (Oxford Immunotec Ltd, Abingdon, UK) which assesses by enzyme-linked immunospot (ELISPOT) the number of blood cells releasing IFN-γ. The antigens used in QFT-GIT (ESAT-6, CFP-10 and TB7.7), are present in *M. tuberculosis* but absent from the BCG vaccine strain and from most NTM, except for *M. kansasii*, *M. marinum*, *M. szulgai*, *M. flavescens*, and *M. gastrii*. Thus, the proteins used as test antigens offer greater specificity compared to TST [13, 14].

Different protocols for LTBI diagnosis combining TST and IGRAs are currently used worldwide but the optimal screening strategy is still debated. In the United States, the Centers for Disease Control and Prevention (CDC, Atlanta, GA) have suggested that IGRAs can be used in place of TST for detection of TB infection [1]. However, there are only limited data on the reproducibility of IGRAs during serial testing. Studies have documented conversions and reversions during repeated IGRAs and there is no consensus on how to define and interpret these phenomena [6, 15–17]. In the United Kingdom, the National Institute for Health and Clinical Excellence (NICE) guidelines recommend a two-step strategy for LTBI diagnosis based on the initial screening with TST followed by an IGRA in TST-positive cases [18, 19].

With an overall TB incidence of 5.7 per 100,000 inhabitants, Italy is considered among those countries at low TB incidence. WHO guidelines indicate that in high-income and upper middle-income countries, with low TB incidence, systematic testing of LTBI, using IGRAs or TST, should be considered for HCWs [3]. However, in countries like Italy where until recently BCG vaccination has been widely used in HCWs, surveillance of TB infection has been hampered by the low specificity of TST. Current Italian guidelines for LTBI screening among HCWs recommend TST as primary assay, that can be integrated with IGRAs in the case of TST positive result [20].

The present work is a retrospective study under a LTBI screening program among HCWs at the Siena University Hospital (Italy) based on the initial screening with TST followed by QFT-GIT assay in TST-positive cases. The agreement between the two tests, the LTBI prevalence among Siena’s hospital HCWs and the identification of putative risk factors for LTBI, were analyzed.

## Methods

A screening program for TB was carried out from September 2011 at the Siena University Hospital that is a low TB risk level hospital. According to this program, all HCWs and students with access to the health profession school, undergo TST as first step examination, followed by QFT-GIT in the case of TST-positive result. TST is repeated yearly in HCWs serving high risk wards, such as Infectious Diseases ward and Microbiology Laboratories.

**Fig. 1:**
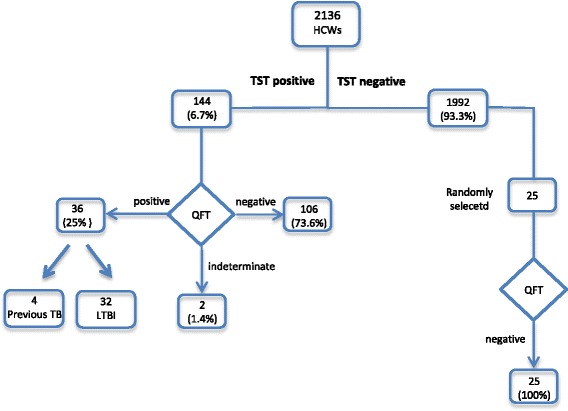
Study population flow chart. HCWs: Healthcare Workers; TST: Tuberculin Skin Test; QFT: Quantiferon; TB: Tuberculosis; LTBI: Latent Tuberculosis Infection

A total of 2136 HCWs were screened from September 2011 to July 2015 by TST. All TST-positive individuals (*n* = 144), as well as a control group of randomly selected subjects with negative TST (*n* = 25), performed QFT-GIT and were enrolled in this retrospective analysis (*n* = 169) (Fig. 1). A group of 142 HCWs was excluded from the study since they were screened only by IGRA because TST was refused (*n* = 64, 45.1 %), contraindicated (*n* = 59, 41.5 %) or avoided for other motivations (*n* = 19, 13.4 %). The time elapsed between TST and blood draw for QFT-GIT was variable. In 30 cases blood for IGRA testing was drawn within 15 days, in 38 cases within 6 months and in 17 within 1 year. In the cases with known positivity to TST in the past, the time elapsed was longer than 1 year. The Fisher exact test showed that there was not a statistically significant correlation between time intervals (TST and blood draw for QFT-GIT) and the probability of a positive QFT-GIT result (*p* = 0.282).

The subjects with positivity for TST and IGRA underwent a chest X-ray to exclude an active TB disease. In the absence of clinical and radiographic signs of active TB, they were considered to have a LTBI and underwent a clinical follow up at 6, 12 and 24 months after diagnosis. An infectious diseases consultant evaluated all LTBI cases and the decision of prescribing prophylaxis was taken case by case.

TST was performed by trained personnel following standard procedures. Intradermal injection of 0.1 ml of PPD (TUBERTEST®, Sanofi Pasteur MSD, France) containing 5 tuberculin units was performed into the volar surface of the forearm. The reaction to TST was assessed after 48 to 72 h from the injection by health-care workers trained to read TST results, while self-reporting of results was not allowed. Induration of 10 mm or more was considered a positive result in persons who had increased risk factors to TB exposure such as HCWs according to CDC’s interpretation criteria [1]. A diameter of 5 mm or more was considered positive in HCWs after accidental contact with infectious patients or materials (identified as “contacts”).

The QuantiFERON®-TB Gold In-Tube test was performed in accordance with the manufactures’s instructions. Briefly, 1 mL of blood was sampled in each of the three tubes containing no antigen (Nil Control tube), TB-specific antigens (ESAT-6, CFP-10 and TB7.7; TB Antigen tube), and positive control (Mitogen Control tube), respectively. Tubes were incubated at 37 °C for 16–24 h before centrifugation, and IFN-γ concentration (IU/ml) was measured by ELISA following the manufacturer’s protocol. The test was considered positive when IFN-γ concentration was ≥ 0.35 IU/ml after correction for the negative control.

For each individual, information on age, gender, place of birth, job category, workplace, previous exposure to TB, family history of TB, prior TST results and BCG vaccination was collected by clinical chart review and vaccination records. BCG vaccination was mandatory in Italy for HCWs until 2001, when it was restricted to contacts and HCWs exposed to multidrug resistant strains with a negative TST at first screening.

According to the retrospective observational nature of the present study and to the authorizations signed by the HCWs related to personal data management including scientific research, formal ethical approval by local Ethics Committee is not requested. Personal information on the subjects included in the study was protected according to Italian law [Italian Law decree n. 196, 30 June 2003 (article 24): http://www.camera.it/parlam/leggi/deleghe/03196dl.htm.].

### Statistical analysis

The concordance between the TST and QFT-GIT results was measured by kappa (k) statistics. A k value ≤ 0.4 was regarded as poor, > 0.8 as excellent, and in between as fair to good agreement according to the Landis and Koch scale. The Fisher exact test was used to compare the frequencies of test results among different groups of participants. Descriptive statistics were reported as mean values ± standard deviations for quantitative variables and as frequencies and percentages for qualitative variables. A *p* value ≤ 0.05 was considered statistically significant. All statistical analysis were performed by using GraphPad 6 software.

## Results

Of the 2136 screened subjects, 1992 resulted TST-negative (93.3 %) and 144 TST-positive (6.7 %) using the established cut-off values. QFT-GIT was performed in all TST-positive subjects, as well as in 25 randomly selected HCWs with a negative TST. Of the 144 TST-positive HCWs, 36 were QFT-GIT positive (25 %), 106 negative (73.6 %) and two indeterminate (1.4 %) therefore excluded from our analysis (Fig. 1). Selected subjects with negative TST were all negative to the QFT-GIT test (Fig. 1). Among the 36 cases of TST+/QFT-GIT+, 4 had radiological signs of previous TB disease (0.2 % of the total HCWs evaluated) while 32 were diagnosed as LTBI (1.5 % of the total HCWs evaluated). Chest X-rays excluded an active TB disease in all cases of positivity for both TST and IGRA. All LTBI cases were evaluated by an infectious diseases consultant but in any case a chemoprophylaxis was indicated. None of the TST positive cases developed active TB in the following months/years.

Overall agreement between IGRA and TST was poor (*k* = 0.092; 95 % Confidence Interval [CI]- 0.048–0.136, *p* = 0.002) (Table 1). Discordance between the two tests was significantly higher among BCG-vaccinated (86.3 %) than unvaccinated subjects (52.8 %) (*p* < 0.001), regardless of time since vaccination (Table 2). As shown in Table 2, more than 50 % of the HCWs had reported a history of BCG vaccination, while 25.3 % had never received BCG. Nevertheless, a clear history of BCG vaccination could not be retrieved in 23.3 % of the studied population.

**Table 1:** TST and IGRA results

	IGRA				
TST	Negative n. (%)	Positive n. (%)	Total n. (%)	*k*	*p-*value
Negative	25 (15.0)	-	25 (15.0)	0.092^b^	0.002
Positive	106 (63.5)	36 (21.6)	142 (85.0)^a^		
All	131 (78.4)	36 (21.6)	167 (100)		

^a^Two IGRA-indeterminate subjects were excluded

^b^95 % CI- 0.048–0.136

**Table 2:** Association of TST and QFT-GIT results with demographic and occupational characteristics of TST-positive HCWs population

Characteristics	Total n. (%)	TST/IGRA		*p*-value
		+/+ n. (%)	+/− n. (%)	
Female	97 (68.3)	22 (22.7)	75 (77.3)	0.304
Male	45 (31.7)	14 (31.1)	31 (68.9)	
Age, years				
19–29	2 (1.4)	0 (0.0)	2 (100.0)	0.272
30–39	40 (28.2)	8 (20.0)	32 (80.0)	
40–49	50 (35.2)	10 (20.0)	40 (80.0)	
50–59	43 (30.3)	15 (34.9)	28 (65.1)	
≥ 60	7 (4.9)	3 (42.9)	4 (57.1)	
Place of birth				
High endemic	14 (9.8)	4 (28.6)	10 (71.4)	0.752
Low endemic	128 (90.2)	32 (25.0)	96 (75.0)	
BCG vaccination				
yes	73 (51.4)	10 (13.7)	63 (86.3)	<0.001
no	36 (25.3)	17 (47.2)	19 (52.8)	
unknown	33 (23.3)	9 (22.3)	24 (72.7)	
Time since BCG vaccination, years				
< 10	3 (4.1)	1 (33.3)	2 (66.7)	0.184
10–14	13 (17.8)	3 (23.1)	10 (76.9)	
≥ 15	57 (40.1)	6 (10.5)	51 (89.5)	
Previous TST				
yes	95 (66.9)	23 (24.2)	72 (75.8)	0.685
no	47 (33.1)	13 (27.6)	34 (72.4)	
TB contacts				
yes	32 (22.5)	7 (21.8)	25 (78.2)	0.817
no	110 (77.5)	29 (26.4)	81 (73.6)	
Job category				
Physician	15 (10.6)	4 (26.7)	11 (73.3)	0.555
Nurse	66 (46.5)	13 (19.7)	53 (80.3)	
Nurse assistant	29 (20.4)	8 (28.6)	20 (71.4)	
Student	12 (8.5)	4 (33.3)	8 (66.7)	
other	20 (14.0)	7 (35.0)	13 (65.0)	
Ward				
High TB risk	15 (10.6)	3 (20.0)	12 (80.0)	0.760
Low TB risk	127 (89.4)	33 (26.0)	94 (74.0)	

No association between other occupational and demographic factors and test results was observed. Indeed, gender (68.3 % were female), age (median age 44.4 years), birth in high (9.8 %) or low (90.2 %) endemic places, working in high TB risk wards (10.6 %), previous exposure to TB infectious patients or materials (22.5 %), and job category (most of HCWs were nurses) did not represent putative risk factors for a positive QFT-GIT test (Table 2).

The probability of a QFT-GIT positive result increased with the TST diameter (*p* = 0.001; Table 3). Indeed, HCWs with a TST <10 mm were all QFT-GIT negative, while in those with a TST between 10 and 15 mm the probability was 24.0 % and in those with TST diameter >15 mm, it was 27.5 % (Table 3).

**Table 3:** Correlation between TST diameter (mm) and QFT-GIT results*

	IGRA				*p*-value
	Negative		Positive		
	n.	%	n.	%	
TST diameter (n.)					
0–9 (29)	29	100.0	-	-	0.001
10–15 (75)	57	76.0	18	24.0	
> 15 (51)	37	72.5	14	27.5	

*p-value refers to the Fisher exact test to compare the frequency distribution of positive and negative QFT-GIT for three increasing diameter ranges

## Discussion

Screening programs for LTBI among HCWs together with TB infection-control measures represent an important tool to reduce the risk of TB transmission in healthcare settings. These screening strategies are also recommended in low TB incidence countries, such as Italy, where an annual TB incidence of 5.7 per 100,000 inhabitants has been estimated [3]. Only a few studies have been conducted to assess the outcome of a two-step LTBI screening strategy (TST plus IGRA) among healthcare workers in Italy [21, 22]. The present work shows the results of a two-step LTBI screening among HCWs in Siena University Hospital and provides an updated epidemiology of LTBI among Italian HCWs. In our setting, a global prevalence of LTBI of 1.5 % was observed, consistent with other studies performed among HCWs in areas with a low TB incidence (mean value of 1 %, range 0.2–12 %) [8, 11, 23]. These data are justified by the low circulation of *M. tuberculosis* in hospitals of low TB prevalence countries that reduces the risk of contact with infected patients.

In our study, 144 subjects were positive to the tuberculin test, corresponding to the 6.7 %. In line with data reported by other Italian and European studies [24–26], only the 22.2 % of TST positive HCWs also had a positive QFT-GIT result. Our data also show that the probability of a QFT-GIT positive result increased with TST diameter and this correlation was already reported in previous studies among HCWs [24, 27, 28].

A poor agreement was observed between the two tests and discordant results (TST+/QFT-GIT-) occurred primarily among BCG vaccinated subjects in line with what was reported in other studies conducted in low TB prevalence countries [25, 29]. This is particularly relevant for countries like Italy where BCG vaccination was mandatory for HCWs until 2001, when it was restricted to defined high TB risk categories only, based on risk assessment at hospital level [30]. Of course, since the sensitivity of QFT-GIT in infected individuals is not 100 %, caution should be taken into account when interpreting discordant results (TST+/QFT-GIT-) occurring in high risk HCWs [14, 18]. Another putative cause of TST+/QFT-GIT- results could be the exposure to NTM [31]. These data strongly support the added value of using both clinical tests, rather than TST alone, for LTBI diagnosis.

## Conclusion

Our data strongly support the added value of using QFT-GIT test in TST-positive HCWs for LTBI diagnosis, especially in settings with a high rate of BCG vaccination. TST is widely used as a first LTBI screening tool but the introduction of IGRA as a second step assay in TST-positive HCWs reduces the potential occurrence of “false positives” due to BCG vaccination or atypical mycobacteria infections and improves the clinical management of LTBI cases. In fact, the introduction of QFT-GIT test could reduce the number of unnecessary chest X-ray and avoid the costs and toxicity associated with unnecessary treatment. Despite the monocentric and observational nature of the study, our results could help to better understand factors influencing screening results and the optimal strategy for LTBI diagnosis among HCWs. Absence of a true gold standard test for LTBI represents a major challenge for determining the accuracy of new LTBI tests, therefore the choice of using a two-step strategy is also aimed at increasing the accuracy level of the diagnostic workup, providing additional information for management and clinical follow-up of LTBI cases.

## Abbreviations

BCG, Bacillus Calmette-Guerin; HCWs, healthcare workers; IGRAs, Interferon gamma release assays; LTBI, latent tuberculosis infection; NTM, non-tuberculous mycobacteria; PPD, purified protein derivative; QFT-GIT -GIT, QuantiFERON-TB Gold In-Tube test; TB, tuberculosis; TST, tuberculin skin test
